# Cytoplasmic SALL4-A isoform expression as a diagnostic marker of less aggressive tumor behavior in gastric cancer

**DOI:** 10.1186/s12957-025-03681-w

**Published:** 2025-02-04

**Authors:** Saeed Rahmani, Amirhesam Babajani, Maryam Abolhasani, Roya Ghods, Elham Kalantari, Zahra Madjd

**Affiliations:** 1 Oncopathology Research Center, Iran University of Medical Sciences, Tehran, Iran; 2 Department of Pathology, School of Medicine, Iran University of Medical Sciences, Tehran, Iran; 3 Department of Molecular Medicine, Faculty of Advanced Technologies in Medicine, Iran University of Medical Sciences, Tehran, Iran

**Keywords:** Gastric cancer, SALL4-A, Biomarkers, Immunohistochemistry, Cancer stem cell

## Abstract

**Background:**

Gastric cancer (GC) poses significant challenges globally, ranking fifth in incidence and fourth in cancer-related mortality. SALL4, a stem cell transcription factor with multiple isoforms, includes SALL4-A as its full-length form. This study aims to evaluate the diagnostic potential of SALL4-A isoform expression in GC and its clinical significance.

**Method:**

Immunohistochemical (IHC) analysis was conducted on Tissue Micro Array (TMA) slides from 167 GC patients. Clinicopathological parameters were correlated with SALL4-A expression, and survival analysis was performed. Diagnostic performance was assessed using metrics such as sensitivity, specificity, and area under the curve (AUC).

**Results:**

SALL4-A exhibited distinct cytoplasmic expression in GC, correlating with lower histological grade (*p* = 0.003) and TNM stage (*p* = 0.003), particularly in the intestinal subtype. Diagnostic evaluation showed an AUC of 0.803 for cytoplasmic expression, demonstrating high diagnostic potential. However, SALL4-A expression did not show significant prognostic value.

**Conclusion:**

Cytoplasmic SALL4-A expression in GC is associated with less aggressive tumor phenotypes and shows promise as a diagnostic marker. Further research is warranted to elucidate its mechanistic role and potential integration into clinical practice.

## Introduction

Gastric cancer (GC) ranks as the fifth most prevalent cancer globally, representing 5.6% of all newly diagnosed cancer cases, exceeding one million instances in 2020. It stands as the fourth leading cause of cancer-related fatalities, contributing to 7.7% of cancer-related deaths [1].

Gastric cancer encompasses various types, each characterized by distinct features and behaviors. The most common type is adenocarcinoma, accounting for 95% of gastric cancer cases [2]. Another subtype is signet ring cell carcinoma, characterized by cells with a distinctive signet ring appearance. Less common types include mucinous adenocarcinoma, which is marked by the presence of mucin-producing cells, and gastrointestinal stromal tumors (GISTs), which originate from the interstitial cells of Cajal [2, 3].

In the past few years, as our understanding of tumor biology and biomarkers has advanced, the approach to cancer treatment has shifted from a generic, one-size-fits-all model with conventional cytotoxic chemotherapy agents to personalized therapies driven by biomarkers. Biomarkers play a crucial role in the management of GC by aiding in identifying optimal treatment strategies and predicting clinical outcomes [4]. In contrast to advancements in biomarker approaches for other cancers, the progress in this area for GC has been comparatively slower. GC's complexity, characterized by its heterogeneous nature with diverse histological and genomic subtypes, poses a challenge in demonstrating diagnostic approaches in clinical trials [5]. Therefore, research endeavors to translate GC's molecular intricacies have become paramount in improving diagnosis, treatment, and patient outcomes.

Recently, a specific transcription factor has emerged as a potential pivotal player in cancer pathogenesis. The Spalt-like (*SALL*) gene family, comprising four members in mammals (*SALL1* to *SALL4*), has gained prominence due to its integral role in embryonic development, contributing significantly to the formation of diverse tissues and organs [6]. Among these, *SALL4*, characterized by a zinc-finger domain and a nuclear localization signal, has attracted considerable attention [7, 8]. *SALL4* is renowned for its crucial involvement in maintaining pluripotency in embryonic stem cells, functioning as a transcriptional regulator that governs gene expression during self-renewal and differentiation processes. Beyond embryonic development, ongoing research has unraveled the multifaceted functions of *SALL4* in various cellular contexts. This transcription factor exerts regulatory control over diverse biological processes, including cell cycle progression, apoptosis, and DNA repair mechanisms [9]. Notably, *SALL4* exists in two distinct isoforms: SALL4-A, derived from the complete transcript, and SALL4-B, a spliced isoform lacking a portion of exon 2. The SALL4 protein features multiple zinc finger cluster (ZFC) domains distributed across different regions, conferring dual functionality in transcriptional activation and repression [10].

Abnormal expression of SALL4 has been reported in various malignancies, such as testicular germ cell, breast, endometrial, lung, liver, and brain cancers, underscoring its potential oncogenic role [11–19]. Earlier investigations have revealed that SALL4 is linked to a poorer prognosis in GC [16, 20]. Nevertheless, the relationship between isotype-specific SALL4 protein expression and clinical outcomes in GC patients remains undefined, and the functional role of SALL4 in GC is yet to be elucidated.

In light of the ongoing ambiguity surrounding SALL4's role in cancer, our study specifically aimed to elucidate the expression and significance of the SALL4-A isoform in gastric tumors. While previous research conducted at our center has explored SALL4-A in testicular cancer [19], no studies have specifically evaluated SALL4-A protein expression or its association with clinicopathological characteristics in gastric cancer or other cancers, highlighting a critical gap in the literature. As the full-length isoform of the SALL4 family, SALL4-A necessitates dedicated investigation; thus, we developed a novel monoclonal antibody specific to SALL4-A, filling the void left by the absence of a commercial antibody [21]. This advancement enabled us to assess isoform-specific expression through immunohistochemistry in tumor and normal specimens from 167 gastric cancer patients. Our primary objective was to clarify the impact of SALL4-A on gastric cancer behavior, with the findings from this study potentially paving the way for improved diagnostic and therapeutic strategies that specifically target SALL4-A, offering a more tailored approach to gastric cancer management.

## Method and materials

### Patient’s characteristics and sample collection

In this examination involving a cross-sectional analysis, we amassed a total of 167 tissue specimens that were formalin-fixed and paraffin-embedded (FFPE) from patients diagnosed with GC. These samples were sourced from the Firoozgar University-affiliated referral hospital in Tehran, Iran, covering the period from 2010 to 2021.

To gain a comprehensive understanding of the clinical and pathological characteristics, we acquired Hematoxylin and Eosin (H&E) stained slides alongside archival medical records. This allowed us to extract key parameters, including patient demographics (age and gender), and clinical details such as maximum tumor diameter, histological grade, TNM stage, presence of distant metastasis, tumor recurrence, and lymphovascular invasions. Only patients who had undergone surgery without prior chemotherapy or radiotherapy were included in the study; however, all patients received chemotherapy post-surgery according to standard guidelines, so it was not analyzed as a variable. Additionally, we included data from 25 adjacent non-malignant tissues to compare the expression of SALL4-A in cancerous samples.

In assessing patient outcomes, we meticulously monitored disease-specific survival (DSS), measured from the time of surgery to the date of the patient's demise related to their tumor. Furthermore, we documented progression-free survival (PFS), defined as the period from the initial surgical intervention to the last follow-up appointment where there was no evidence of disease, metastasis, or recurrence. Our approach to tumor staging adhered to the TNM classification for GC [22]. It is essential to note that this study received ethical approval (Code: IR.IUMS.REC.1401.984) from the Research Ethics Committee of the Iran University of Medical Sciences.

### Tissue microarray (TMA) construction

The construction of GC tissue TMA blocks was carried out according to previously established procedures [23]. Three of the most representative tumor areas from different sections of each block were meticulously identified and marked, ensuring precise alignment with corresponding H&E slides. Subsequently, a precision arraying instrument, specifically the Tissue Arrayer Minicore by ALPHELYS in Plaisir, France, was utilized to extract samples from the designated tumor regions in each block. These samples were then transferred into a new recipient paraffin block. The finalized TMA blocks were sectioned at a thickness of 4-μm and placed on adhesive slides.

TMA blocks were created in triplicate from each GC specimen to address the substantial concern of tumor heterogeneity and enhance the accuracy and validity of data analysis. Previous validation studies for TMA indicated that, even in the presence of variations in antigen expression among individual cores, the analysis of each core effectively captured over 90% of the staining pattern exhibited by the entire tissue section. Moreover, when two readable cores were analyzed, the accuracy exceeded 95% [24–26]. The final score was determined by calculating the mean scores from the three cores. Notably, to compare the expression patterns of SALL4-A with GC tissue specimens, adjacent non-malignant tissue samples were also incorporated into each TMA block [27].

### Immunohistochemistry (IHC) staining

The expression of SALL4-A protein was evaluated via Immunohistochemistry (IHC) on TMA sections using the EnVision-HRP kit (Dako, Glostrup, Denmark) [19, 27, 28]. Briefly, TMA slides were dewaxed by heating at 60°C for 20 min, then cleared of paraffin using xylene, and rehydrated through immersions in graded ethanol. To block endogenous peroxidase activity, slides were incubated in 3% H2O2 in methanol for 15 min in a dark area at room temperature (RT). Antigen retrieval was performed by autoclaving the slides in Tris–EDTA buffer (pH 9) at 95°C for 10 min.

Following antigen retrieval, sections were incubated in 5% normal goat serum diluted in a protein block (Dako, CA, USA) at room temperature. The primary anti-SALL4-A monoclonal antibody (2 μg/ml) [21], was then applied to the tissue sections and incubated for 1 h at room temperature. An affinity-purified non-immune mouse IgG (ARI, Tehran, Iran) was used as the negative reagent control. Slides were then treated with the secondary antibody, anti-rabbit/anti-mouse EnVision (Dako, Glostrup, Denmark), for 40 min at RT. To visualize positive signals, 3,3′-diaminobenzidine (DAB, Dako, Denmark) was applied to the slides for 5 min. The sections were subsequently counterstained with hematoxylin (Dako, Denmark) for 3 min. Finally, the slides were dehydrated through ethanol dilutions, cleared with xylene, and mounted in Entellan mounting medium (Merck, Darmstadt, Germany). Human testicular cancer whole tissue sections were used as positive control tissue for evaluating SALL4-A protein expression.

### Virtually digitizing TMA slides

To ensure a standardized evaluation of the TMA samples, the stained slides were scanned using the Basler ace slide scanner (Basler AG, Germany), allowing for high-resolution digital imaging. Following scanning, the TMA cores were segmented into randomly assigned, concealed-label images to facilitate an unbiased scoring process and prevent observer bias. Image quality and resolution across all TMA core samples were then standardized using the Microvisioneer manualWSI 2022A-1b software (Microvisioneer GmbH, Germany), which provided consistent adjustment of image parameters such as brightness, contrast, and sharpness, ensuring uniform quality for subsequent analysis.

### Evaluation of immunostaining and scoring score

The assessment of SALL4-A expression on Tissue Microarray (TMA) slides underwent a thorough examination led by an expert pathologist (M.A.) along with two trained M.Ds (S.R and A.B) who collaborated to achieve consensus in cases of disagreements. This evaluation employed a semiquantitative scoring system, and the pathologists performed their assessments without access to the clinicopathological and survival data of the patients. The Aperio ImageScope 12.4.6 software served as the tool for viewing TMA core images.

The examination of SALL4-A expression involved scrutinizing three distinct scoring criteria: staining intensity, the proportion of positive tumor cells, and the H-score. Staining intensity received a score on a 4-point scale, differentiating between negative or non-staining (scored as 0), weak (scored as 1), moderate (scored as 2), and strong (scored as 3). The Histochemical score (H-score) was calculated by multiplying the staining intensity and the percentage of positive cells, resulting in a score ranging from 0 to 300 for each case [29]. Subsequently, the H-scores were categorized into two groups based on the mean value: low expression (≤ mean) and high expression (> mean).

### Statistical analysis

All statistical analyses were conducted using version 25.0 of the SPSS statistical software from SPSS, Inc., an IBM Corp company based in the USA. Categorical data were represented as N (%), while quantitative data were presented as either mean (SD) or median (Q1, Q3). Significance testing for associations and correlations between SALL4-A protein expression and clinicopathological characteristics utilized Pearson's chi-square and Spearman's correlation tests. Pairwise comparisons between study groups were conducted using Kruskal–Wallis and Mann–Whitney U tests.

Survival analysis involved generating survival curves through the Kaplan–Meier method, with a 95% confidence interval (CI). The log-rank test was employed to compare survival outcomes between groups with low and high marker expression. The univariate Cox proportional hazards regression model was utilized to assess variables impacting Disease-Specific Survival (DSS) or Progression-Free Survival (PFS). Variables demonstrating a significant impact on survival in the univariate analysis were included in multivariable Cox proportional hazards regression analyses.

To evaluate the diagnostic value of the SALL4-A protein, receiver operating characteristic (ROC) curves were analyzed, and the area under the ROC curve (AUC), sensitivity, and specificity were calculated. Throughout the entire analysis, statistical significance was established at *P* < 0. 05.

## Results

### Characteristics of patients’ tissue samples

This cross-sectional study involved the enrollment of a total of 167 tissue specimens. Within this cohort, 63 samples (37.7%) were identified as signet ring cell carcinoma, and 104 samples (62.3%) were classified as the intestinal type. The mean age of the entire sample set was 61.1 years, with ages ranging from 24 to 84 years. In terms of gender distribution, 124 (74.25%) were male and 43 (25.75%) were female. The histological grade was categorized as well-differentiated, moderately differentiated, and poorly differentiated [30]. All clinicopathological features of our total samples and subtypes of GC are described in Table 1.

**Table 1:** Patients and tumor clinicopathological characteristics of gastric cancer and its histological subtypes

**Patients and tumor characteristics**	**Total samples N (%)**	**Signet Ring Cell Carcinoma N (%)**	**Intestinal type N (%)**
**Number of samples**	167	63 (27.0)	104 (44.6)
**Mean age, years (Range)**	61.13 (24–84)	54.95 (24–79)	64.88 (27–84)
≤ Mean age	76(49.62)	28 (44.4)	48 (46.2)
> Mean age	91 (70.07)	35 (55.6)	56 (53.8)
**Gender**			
Male	124 (74.25)	41 (65.1)	83 (79.8)
Female	43 (25.75)	22 (34.9)	21 (20.2)
**Mean tumor size (cm) (Range)**	4.9 (1–15)	5.26(1–15)	4.69(1–13)
≤ Mean	80 (50.6)	33 (58.9)	52(53.1)
> Mean	78 (49.4)	23 (41.1)	46 (46.9)
**Histological grade**			
Well-differentiated	38 (24.05)	2 (3.2)	36 (37.5)
Moderate differentiated	37 (23.41)	-	37 (38.5)
Poor differentiated	83 (52.53)	60 (96.8)	23 (24)
**TNM stage**			
I	36 (22.78)	13 (21.7)	23 (23.5)
II	50 (31.64)	16 (26.7)	34 (34.7)
II I	64 (40.5)	26 (43.3)	38 (38.8)
IV	8 (5.06)	5 (8.3)	3 (3.1)
**Tumor site**			
Cardia	15 (9.03)	4 (6.5)	11 (10.6)
Fundus	2 (1.2)	-	2 (1.9)
Body	18 (10.84)	7 (11.3)	11 (10.6)
Antrum	35 (21.08)	12 (19.4)	23 (22.1)
Pylors	9 (5.42)	4 (6.5)	5 (4.8)
Non-Specified	87 (52.40)	35 (56.5)	52 (50)
**Lymphovascular invasion**			
Present	69 (44.23)	26 (45.6)	43 (43.4)
Absent	87 (55.76)	31 (54.4)	56 (56.6)
**Tumor recurrence**			
Yes	27 (17.64)	9(15.8)	18 (18.8)
No	126 (82.35)	48(84.2)	78 (81.3)
**Distant metastasis**			
Yes	59 (38.56)	24 (42.1)	35 (36.5)
No	94 (61.43)	33 (57.9)	61 (63.5)

### Expression of SALL4-A in GC compared with adjacent non-malignant samples

**Fig. 1:**
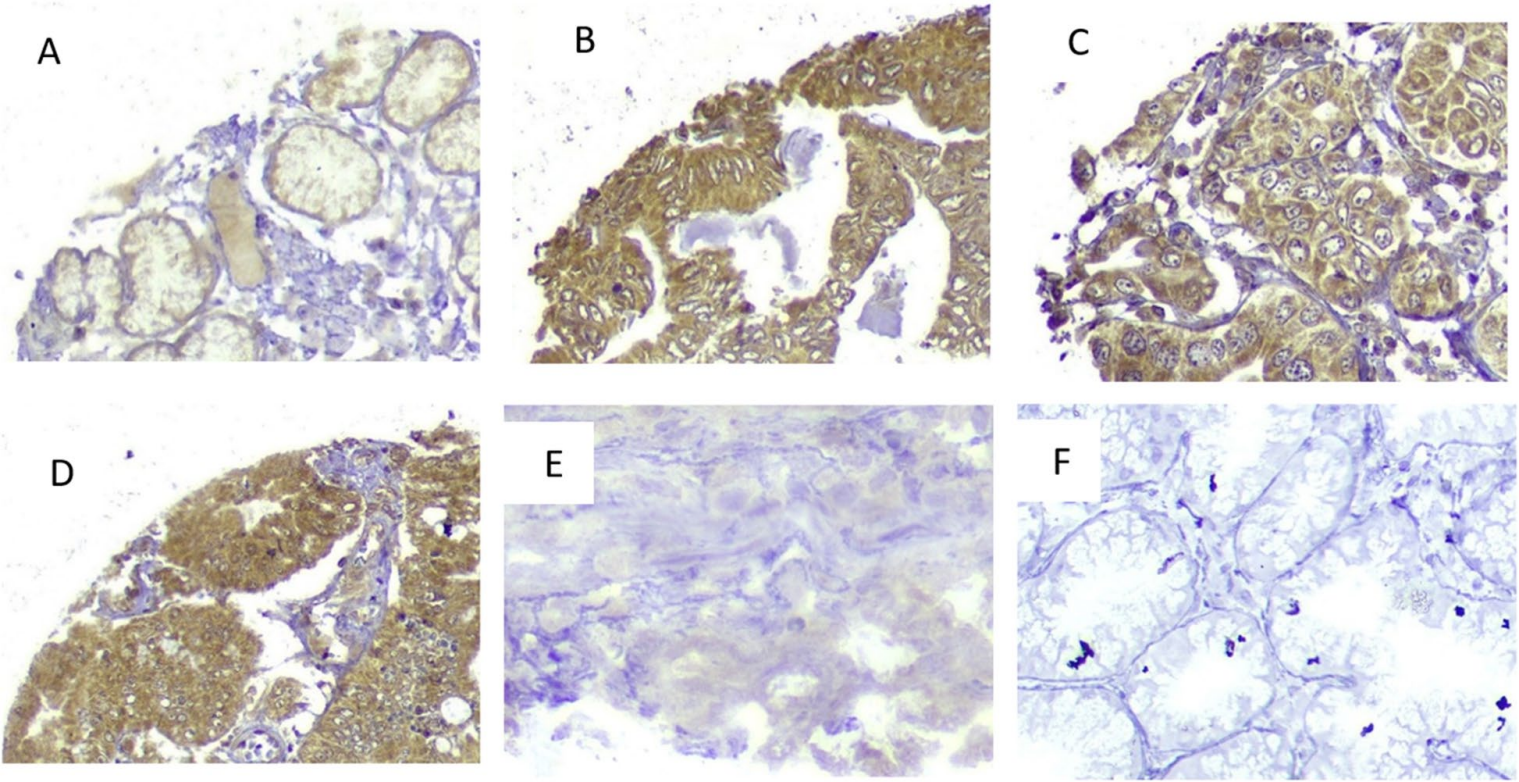
TMA core stained by the monoclonal anti-SALL4-A antibody (40 × magnification). Immunohistochemical (IHC) staining demonstrates varied SALL4-A expression in the cytoplasmic and nuclear compartments of gastric cancer cells. **A** Predominantly low cytoplasmic expression of SALL4-A. **B** Predominantly high cytoplasmic expression of SALL4-A. **C** Low nuclear expression of SALL4-A. **D** High nuclear and cytoplasmic expression of SALL4-A. **E** Non-malignant normal gastric tissue with low SALL4-A expression. **F** Isotype control tissue, showing no specific staining

IHC was utilized to evaluate SALL4-A protein expression in TMA sections of gastric tumors, employing three distinct scoring methods: staining intensity, the proportion of positive tumor cells, and the H-score. SALL4-A demonstrated varying expression levels in both the nucleus and cytoplasm within the two major subtypes of GC (Fig. 1).

The mean expression levels of SALL4-A, as determined by H-score, were markedly higher in tumoral cores compared to adjacent non-malignant tissues. In tumoral samples, nuclear expression levels averaged 102.11 ± 84.99, while cytoplasmic expression levels reached 158.51 ± 65.83. In contrast, the adjacent non-malignant tissues showed significantly lower mean expression levels, with nuclear scores of 50.8 ± 49.05 and cytoplasmic scores of 90.5 ± 55.2 (Table 2). Statistical analysis using the Mann–Whitney U test further confirmed significant differences in SALL4-A expression between malignant and adjacent non-malignant samples, both in the nucleus (*P* = 0.01) and cytoplasm (*P* = 0.00). These results underscore a notable increase in SALL4-A expression within cancerous tissues, suggesting a potential role of this protein in tumorigenesis or tumor behavior.

**Table 2:** Association of nuclear and cytoplasmic SALL4-A protein expressions (Intensity of staining, percentage of positive tumor cells, and H-score) between subtypes of gastric cancer and benign tumors

**Expression of SALL4-a**	**Nuclear expression**			
	**Total samples N(%)**	**Signet Ring Cell Carcinoma N (%)**	**Intestinal type N (%)**	**Adjacent Normal Tissue N (%)**
**Intensity of Staining**				
Negative (0)	26 (15.56)	12 (19.0)	14 (13.5)	9 (36)
Weak (+ 1)	30 (17.96)	14 (22.2)	16 (15.4)	13 (52)
Moderate (+ 2)	59 (35.32)	22 (34.9)	37 (35.6)	3 (12)
Strong (+ 3)	52 (31.13)	15 (23.8)	37 (35.6)	0
**Percentage of positive tumor cells**				
< 25%	50 (29.94)	20 (31.7)	30 (28.8)	10 (40)
25–50%	46 (27.54)	15 (23.8)	31 (29.8)	5 (20)
51–75%	40 (23.95)	17 (27)	23 (22.1)	5 (20)
> 75%	31 (18.56)	11 (17.5)	20 (19.2)	5 (20)
**H-score Mean**	100	98.9	104	44.7
Low	91 (54.49)	33 (52.3)	58 (55.7)	13 (52)
High	76 (45.51)	30 (47.7)	46 (44.3)	12 (48)
**Expression of SALL4-a**	**Cytoplasmic expression**			
	**Total samples N(%)**	**Signet Ring Cell Carcinoma N (%)**	**Intestinal type N (%)**	**Adjacent Normal Tissue N (%)**
**Intensity of staining**				
Negative (0)	2 (1.19)	2 (3.2)	0	4 (16)
Weak (+ 1)	39 (23.35)	25 (39.7)	14 (13.5)	13 (52)
Moderate (+ 2)	65(38.92)	20 (31.7)	45 (13.5)	8 (32)
Strong (+ 3)	61 (36.52)	16 (25.4)	45 (43.3)	0
**Percentage of positive tumor cells**				
< 25%	3 (1.79)	3 (4.8)	0	3 (12)
25–50%	12(7.18)	7 (11.1)	5 (4.8)	1 (4)
51–75%	70 (41.91)	24 (38.1)	46 (44.2)	13 (52)
> 75%	82 (49.10)	29 (46)	53 (51)	8 (32)
**H-score Mean**	158.6	133.3	173.7	88.5
Low	98 (58.7)	37 (58.7)	61 (58.7)	14 (56)
High	69 (41.3)	26 (41.3)	43 (41.3)	11 (44)

*H-score* Histological Score

**Fig. 2:**
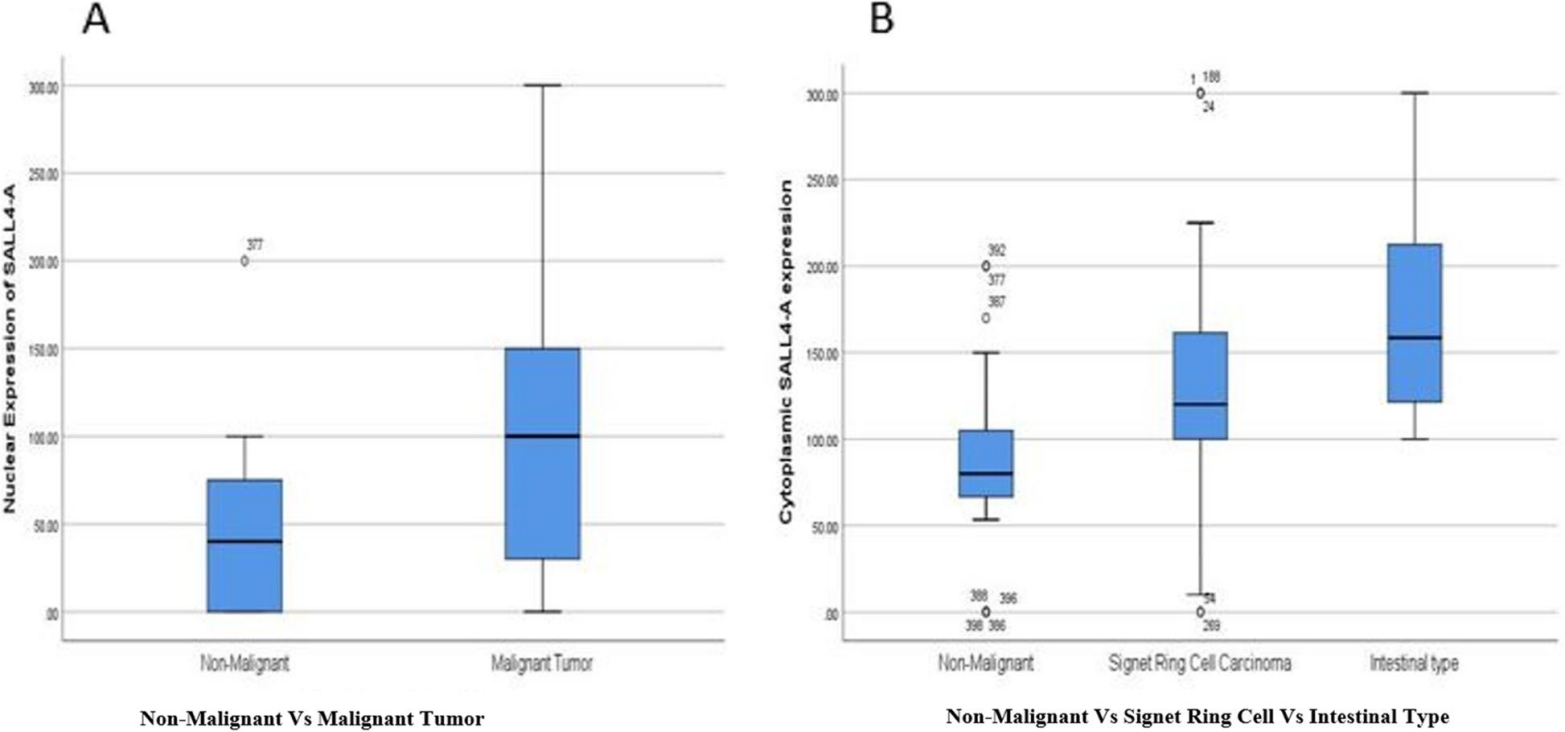
Nuclear and Cytoplasmic expression of SALL4-A among different sample types of GC.** A** There is a significant difference in the Nuclear expression of SALL4-A between malignant and non-malignant adjacent tissue (*P* = 0.01). **B** There is a significant difference in Cytoplasmic expression of SALL4-A between normal and malignant tissue (*P* = 0.00) and the GC subtypes (*P* = 0.01)

Also, Mann–Whitney U tests indicated a significant difference in cytoplasmic expression (but not nuclear) of SALL4-A between Signet Ring Cell Carcinoma and Intestinal subtypes (*P* = 0.001) (Fig. 2).

### Associations between SALL4-A expression and clinicopathological features of GC

The association between SALL4-A expression and clinicopathological parameters in gastric tumors, encompassing both Signet Ring Cell Carcinoma and Intestinal types, was investigated using Pearson's χ2 test. Our analysis uncovered a notable correlation between cytoplasmic SALL4-A protein expression (based on H-score) and specific clinicopathological characteristics, including age (*P* = 0.03), tumor type (*P* = 0.00), histological grade (*P* = 0.00), and TNM stage (*P* = 0.01).

Additionally, Spearman's correlation analysis was employed to explore the association between SALL4-A expression and clinicopathological features. The results revealed a significant inverse correlation between cytoplasmic expression of SALL4-A and parameters such as histological grade (*P* = 0.003) and TNM stage (*P* = 0.003), indicating that higher levels of expression of SALL4 were found more in well-differentiated and lower stage tumors. (Table 3), Meanwhile, Mann–Whitney U tests demonstrated that higher cytoplasmic expression of SALL4-A was correlated with the intestinal type of GC (*P* = 0.001).

**Table 3:** The association between expression of SALL4-A and clinicopathological features of gastric tumors

**Tumor characteristics**	**Total samples N (%)**	**Nuclear expression**		***P value***	**Cytoplasmic expression**		***P value***
		**H score (Mean = **100**) N %**			**H score (Mean = 158.6) N %**		
		**Low (≤ 100)**	**High (> 100)**		**Low (≤ 158.6)**	**High (> 158.6)**	
**Number of samples**	167	135	98		135	98	
**Mean age, years (Range)**	61.13(24–84)			*0.46*			***0.03***
≤ Mean age	76 (45.5)	49	33		55	27	
> Mean age	91 (54.5)	46	39		43	42	
**Gender**				*0.20*			*0.52*
Male	124 (74.25)	67	57		71	53	
Female	43 (25.74)	28	15		27	16	
**Mean tumor size (cm) (Range)** ≤ Mean	4.9 (1–15) 80 (50.6)	50	30		47	33	
> Mean	78 (49.4)	39	35	*0.22*	43	31	*0.93*
**Tumor type**				*0.48*			***0.00***
Signet Ring Cell Carcinoma	63 (27)	41	22		46	17	
Intestinal Type	104 (44.6)	57	47		52	52	
**Histological grade**				*0.37*			***0.00***
Well-differentiated	38 (24.05)	25	13		16	22	
Moderate differentiated	37 (23.41)	19	18		21	16	
Poor differentiated	83 (52.53)	46	37		57	26	
**TNM stage**				*0.12*			***0.01***
I	36 (22.78)	23	13		17	19	
II	50 (31.64)	31	19		26	24	
III	64 (40.5)	34	30		41	23	
IV	8 (5.06)	3	5		7	1	
**Lymphovascular Invasion**				*0.36*			***0.02***
Present	69 (44.23)	37	32		47	22	
Absent	87 (55.76)	53	34		44	43	
**Recurrence**				*0.24*			*0.16*
Yes	27 (17.64)	13	14		13	14	
No	126 (82.35)	76	50		79	47	
**Metastasis**				*0.43*			*0.87*
Yes	59 (38.56)	32	27		35	24	
No	94 (61.43)	57	37		57	37	

*P* value, Pearson's chi-square

Values in bold are statistically significant

*H-score:* histological score

#### Clinical outcomes in subtypes of gastric cancer

In our investigation, which encompassed 167 patients after exclusions, we noted that 27 individuals (17.53%) encountered tumor recurrence, while 59 patients (38.31%) experienced instances of metastasis. Additionally, cancer-related fatalities were documented in 71 individuals (46.1%). 68 patients (44.15%) were alive without distant metastasis and tumor recurrence. 13 patients discontinued their collaboration during follow-up.

The average duration of Disease-Specific Survival (DSS) and Progression-Free Survival (PFS) follow-up were 42.56 months (SD = 28.3) and 39.8 months (SD = 27.68), respectively. A comprehensive summary of the survival outcomes is presented in Table 4.

**Table 4:** The main characteristics of patients’ survival analysis based on subtypes of gastric cancer

**Features**	**Total samples**	**Histological subtypes of GC**	
		**Signet Ring Cell Carcinoma**	**Intestinal type**
Number of patients (N)	154	58	96
Range of follow-up (month)	0–114	0–114	0–108
Mean duration of follow-up time (month) for OS, DSS (SD)			
OS	42.56 (28.3)	41.24 (29.148)	43.36 (27.907)
DSS	42.56 (28.3)	41.24 (29.148)	43.36 (27.907)
Median duration of follow-up time (month) for OS, DSS (Q1, Q3)			
OS	42.50 (17.75–61)	40.00 (18.50, 59.50)	46.00 (18.25, 60.75)
DSS	42.50 (17.75–61)	40.00 (18.50, 59.50)	46.00 (18.25, 60.75)
Mean duration of follow-up time (month) for PFS (SD)	39.8 (27.68)	37.52 (25.815)	41.15 (28.771)
Median duration of follow-up time (month) for PFS (Q1, Q3)	38 (15–59)	37.00 (12.75, 58.00)	41.00 (15, 60)
Cancer-related death (N %)	71 (46.1)	28 (44.4)	43 (41.3)
Death due to other reasons (N %)	8 (5.19)	1 (1.6)	7 (6.7)
Distant metastasis during follow-up (N %)	59 (38.31)	24 (38.1)	35 (33.7)
Tumor recurrence during follow-up (N %)	27 (17.53)	9 (14.3)	18 (17.3)
Alive patients without distant metastasis and tumor recurrence (N %)	68 (44.15)	28 (44.4)	40 (38.5)

### Survival analysis based on the expression of SALL4-A in GC subtypes

**Fig. 3:**
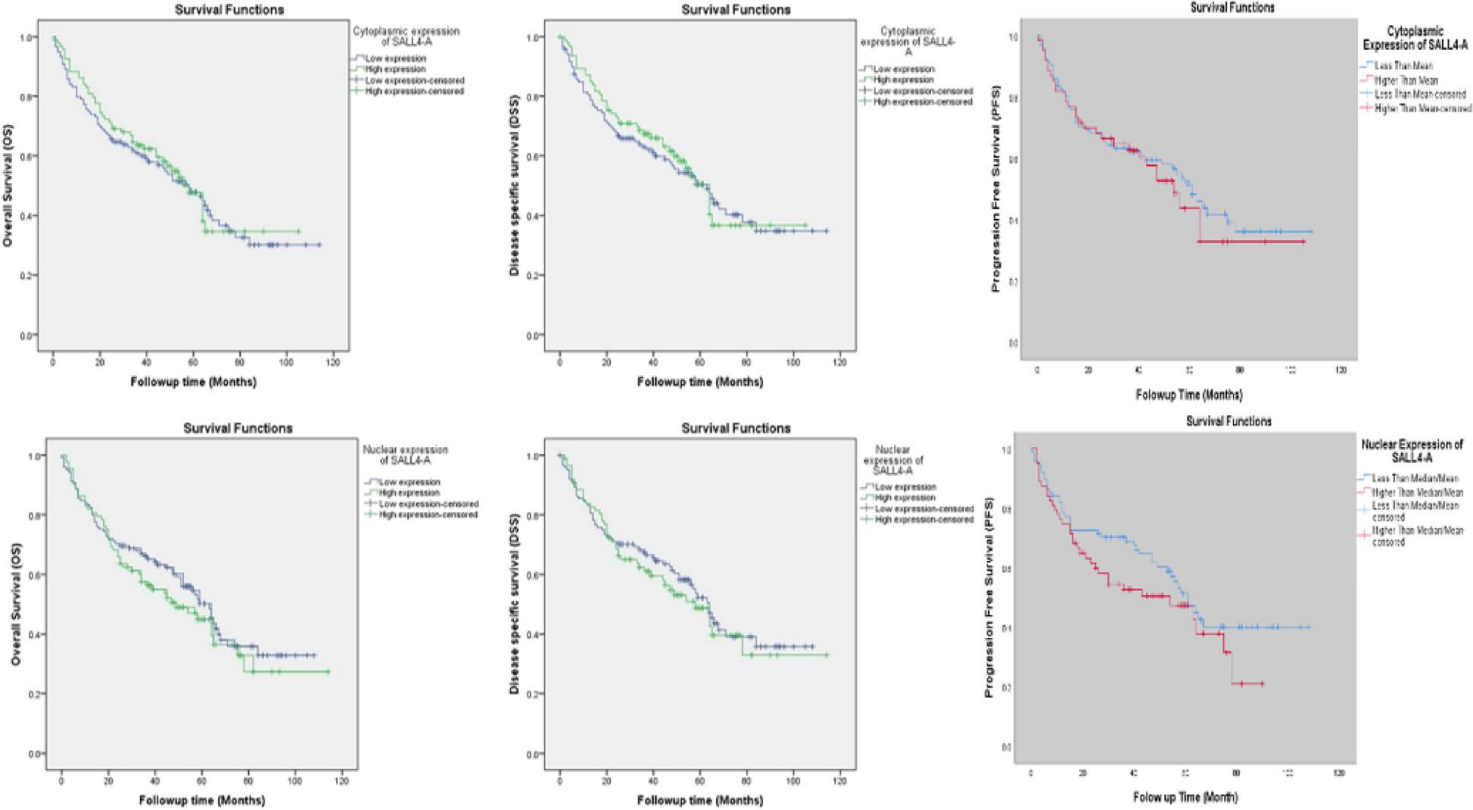
Kaplan–Meier survival analysis of cytoplasmic and nuclear expression of SALL4-A based on H-scores of IHC

The Kaplan–Meier survival analysis did not reveal any statistically significant differences in terms of Survival when comparing patients whose tumors express high and low levels of SALL4-A(H-score) (Fig. 3).

Univariate and multivariate Cox regression analyses were conducted to evaluate the clinical significance of potential prognostic factors for Overall Survival (OS), Disease-Specific Survival (DSS), and Progression-Free Survival (PFS). In the univariate analyses, TNM stage, tumor recurrence, and distant metastasis emerged as potential prognostic factors for OS. Additionally, TNM stage and distant metastasis were identified as potential prognostic factors for DSS. Furthermore, histological grade, distant metastasis, and cytoplasmic expression of SALL4-A were recognized as potential prognostic factors for PFS.

The statistically significant findings from the univariate analyses were subsequently incorporated into the multivariate Cox regression analysis. The results of the multivariate analysis revealed that histological grade (well vs. moderate) [HR: 2.476, 95% CI: (1.062–5.774)] significantly impacted PFS, while TNM (I vs. III) [HR: 1.399, 95% CI: (1.024–1.911) for OS and HR: 1.505, 95% CI: (1.080–2.096) for DSS] and distant metastasis [HR: 0.168, 95% CI: (0.104–0.273) for OS and HR: 0.130, 95% CI: (0.076–0.222) for DSS] were independent risk factors significantly influencing survivals, respectively (Table 5).

**Table 5:** Univariate and multivariate Cox regression analyses of potential prognostic factors for Overall Survival (OS), Disease-Specific survival (DSS), and Progression-Free Survival (PFS) in patients with GC

**Covariate**	**Overall Survival (OS)**				**Disease-Specific survival (DSS)**				**Progression-Free Survival (PFS)**			
	**Univariate analysis**		**Multivariate analysis**		**Univariate analysis**		**Multivariate analysis**		**Univariate analysis**		**Multivariate analysis**	
	**HR (95% CI)**	***P-value***	**HR (95% CI)**	***P-value***	**HR (95% CI)**	***P-value***	**HR (95% CI)**	***P-value***	**HR (95% CI)**	***P-value***	**HR (95% CI)**	***P-value***
**Age (years)**	1.014 (0.998–1.030)	0.079			1.012 (0.996–1.028)	0.155			1.006(0.990–1.024)	0.451		
**Tumor size (cm)**	1.062 (0.994–1.134)	0.074			1.064 (0.993–1.140)	0.076			0.972 (0.899–1.052)	0.481		
**Histological grade**	1.096 (0.866–1.387)	0.447			1.107(0.864–1.419)	0.421			0.913 (0.701–1.188)	0.497	-	-
Well Vs Poor	0.773(0.465–1.286)	0.322			0.740 (0.429–1.277)	0.279			1.325 (0.809–2.171)	0.264	-	-
Well Vs Moderate	0.694 (0.405–1.188)	0.183			0.632 (0.357–1.118)	0.115			2.193 (1.186–4.057)	**0.012**	2.476 (1.062–5.774)	**0.036**
Moderate Vs Poor	1.131 (0.741–1.728)	0.569			1.187 (0.763–1.840)	0.449			0.626 (0.366–1.073)	0.088		
**TNM stage**	1.231(1.002–1.513)	**0.048**			1.257 (1.013–1.560)	**0.038**			1.024(0.817–1.284)	0.835		
I Vs II	0.797(0.480–1.325)	0.382	-	**-**	0.789 (0.460–1.355)	0.391	-	**-**	1.022 (0.616–1.694)	0.933		
I Vs III	0.556(0.341–0.908)	**0.019**	1.399 (1.024-1.911)	**0.035**	0.516 (0.309–0.864)	**0.012**	1.505 (1.080-2.096)	**0.016**	0.938 (0.555–1.586)	0.811		
I Vs IV	0.787 (0.299–2.071)	0.628			0.867 (0.296–2.536)	0.794			1.070 (0.408–2.802)	0.891		
II Vs III	0.695 (0.449–1.075)	0.102	0.551(0.207-1.467)	0.233	0.642 (0.407–1.012)	0.056	0.383 (0.134- 1.098)	0.074	0.942 (0.560–1.586)	0.823		
II Vs IV	1.175 (0.459–3.010)	0.737			1.294 (0.455–3.678)	0.628			1.032 (0.396–2.690)	0.942		
III Vs IV	1.425 (0.563–3.608)	0.455			1.720 (0.614–4.817)	0.302			1.018 (0.384–2.701)	0.972		
**Margin Involvement**	0.984 (0.431–2.250)	0.97			1.057 (0.428–2.607)	0.905			1.054(0.456–2.438)	0.902		
**Lymphovascular invasion**	0.834 (0.568–1.223)	0.352			0.870 (0.581–1.303)	0.499			0.717(0.473–1.089)	0.119		
**Macroscopic tumor extension**	0.981 (0.805–1.195)	0.847			0.955 (0.777–1.175)	0.665			1.100(0.888–1.362)	0.384		
**Tumor recurrence**	0.593 (0.389–0.904)	**0.015**	1.169 (0.688–1.986)	0.563	0.527 (0.344–0.810)	**0.003**	1.110(0.647–1.905)	0.705	0.894(0.448–1.787)	0.752		
**Distant metastasis**	0.171 (0.116–0.254)	**< 0.001**	0.168 (0.104- 0.273)	**< 0.001**	0.135 (0.088–0.208)	**< 0.001**	0.130(0.076–0.222)	**< 0.001**	3.080(1.245–7.622)	**0.015**	1.762 (0.603–5.148)	0.300
**Nuclear SALL4-A expression**												
High versus low	1.183 (0.817–1.712)	0.373	-	-	1.106 (0.75–1.632)	0.61			1.420(0.933–2.163)	0.102		
**Cytoplasmic SALL4-A expression**												
High versus low	1.038 (0.711–1.515)	0.848	-	-	1.070(0.722–1.588)	0.735			1.695(1.129–2.543)	**0.011**	1.761(0.874–3.548)	0.114

The variables with *a P* value less than 0.05 were included in multivariable analyses. Values in bold are statistically significant

*HR* hazard ratio, *CI* Confidence interval

#### Diagnostic value of the SALL4-A in GC versus adjacent non-malignant tissues

**Fig. 4:**
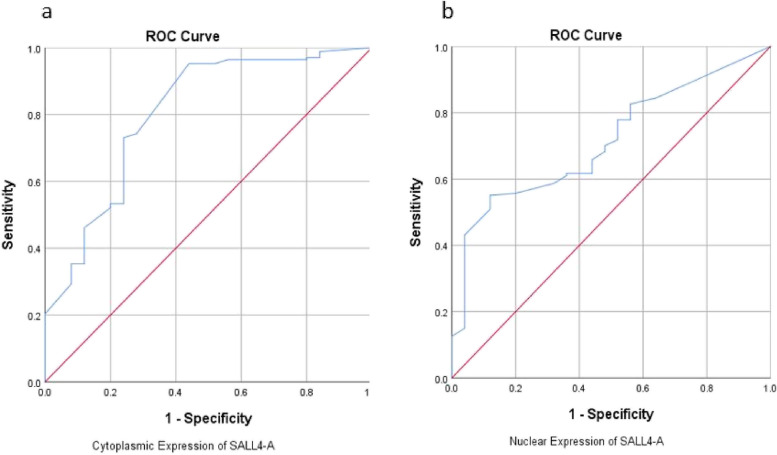
ROC curves and AUC of the SALL4-A protein's expression levels for the diagnostic potential of the SALL4-A (**a**) cytoplasmic and (**b**) nuclear expression levels. The AUC and *P*-values showed significant difference in the diagnostic potential of SALL4-A cytoplasmic and nuclear expression

ROC curves and the calculation of the Area Under the Curve (AUC) were utilized to assess the diagnostic potential of the SALL4-A protein's expression levels, both in the nuclear and cytoplasmic compartments, in distinguishing GC from adjacent non-malignant tissues (Fig. 4). The ROC curve results unveiled an AUC of 0.803 (95% CI: 0.703 – 0.904) and *P*-value of 0.000 for cytoplasmic expression of SALL4-A, AUC of 0.714 (95% CI: 0.622 – 0.807) and *P*-value of 0.001 for nuclear expression of SALL4-A, which showed reliable diagnostic value of expression of SALL4-A for GC (Table 6).

**Table 6:** Diagnostic evaluation of the SALL4-A in gastric cancer patients

**Diagnostic values**	**Nuclear expression**	**Cytoplasmic expression**
**Sensitivity**	55.1%	95.2%
**Specificity**	88%	56%
**PLR**	4.59	2.16
**NLR**	0.604	0.085
**AUC**	0.714	0.803
**Cut-off**	81.5	92.83
***P value***	0.00	0.00

## Discussion

Gastric cancer poses a formidable challenge within the field of oncology, marked by its elevated lethality, late-stage detection, and limited therapeutic avenues. Consequently, there is an imperative demand for enhanced diagnostic and prognostic tools and treatment strategies. Recent efforts have been devoted to identifying potential biomarkers with applications in early diagnosis, prognosis prediction, and assessment of therapeutic responses. Stemness-related biomarkers, in particular, have garnered considerable attention from scientists and clinicians due to their association with the stemness-like attributes of cancer cells and their correlation with the invasive and progressive nature of neoplastic cells [31, 32].

Amid the diverse categories of biomarkers, SALL4 has emerged as a focal point, given its involvement in regulating embryonic stem cells and its pivotal role in cell renewal and proliferation. The intricate regulation of *SALL4* expression encompasses various molecular mechanisms operating at the transcriptional, post-transcriptional, and epigenomic levels [33]. Considering the inherent instability of cancer cells, aberrant *SALL4* expression has been reported in both solid tissues and hematologic malignancies [9, 14–16, 34].

This study highlights the diagnostic potential of the SALL4-A isoform in gastric cancer (GC), emphasizing its association with less aggressive tumor behavior. SALL4-A was predominantly expressed in the cytoplasm of gastric cancer cells, correlating with favorable clinicopathological features, including lower histological grades and early-stage disease. This unique expression pattern distinguishes it from other SALL4 isoforms, which are typically nuclear and associated with tumor aggressiveness in various cancers.

In addition to its own diagnostic potential, SALL4-A complements established gastric cancer (GC) biomarkers such as HER2 and PD-L1 by addressing diagnostic and prognostic gaps. HER2 and PD-L1 are primarily associated with poor-prognosis tumors and serve as therapeutic targets in advanced GC cases. HER2 is widely used to identify patients eligible for trastuzumab therapy, while PD-L1 predicts response to immune checkpoint inhibitors [33, 35]. In contrast, higher cytoplasmic expression of SALL4-A in this study was correlated with favorable clinicopathological features, including lower histological grade, early-stage disease, and the intestinal subtype of GC. These findings position SALL4-A as a promising diagnostic marker, particularly in detecting early-stage or less aggressive tumors, where HER2 and PD-L1 might have limited applicability.

Moreover, the distinct biological roles of these biomarkers underscore their complementary nature. While HER2 and PD-L1 target aggressive disease with therapeutic interventions, SALL4-A’s diagnostic utility lies in identifying less advanced tumors, potentially facilitating earlier detection and intervention. This complementary relationship could enhance GC management by integrating SALL4-A into diagnostic algorithms alongside HER2 and PD-L1, improving accuracy in GC staging and subtype differentiation. Prospective studies are warranted to explore the integration of SALL4-A with multiplex biomarker panels, enabling precise stratification of patients for tailored therapeutic strategies. Additionally, examining the role of SALL4-A in predicting treatment responses, particularly in combination with HER2-targeted therapies or immunotherapy, could further define its clinical utility.

Therapeutic exploration of SALL4-A’s downstream signaling pathways could provide new avenues for targeted interventions. SALL4-A’s role in modulating VEGF-mediated angiogenesis and Wnt/β-catenin pathways highlights its potential as a target for anti-angiogenic therapies and pathway-specific inhibitors [36, 37]. Future studies should explore its integration into diagnostic algorithms alongside HER2 and PD-L1 to refine GC staging and treatment selection.

While SALL4-A showed significant diagnostic potential, particularly in distinguishing between different gastric cancer subtypes, it did not demonstrate a clear prognostic role in this study. This finding suggests that SALL4-A may be more useful as a diagnostic marker, particularly for identifying less aggressive tumor phenotypes, rather than as a prognostic tool for predicting patient outcomes. The distinct cytoplasmic localization in less aggressive gastric tumors observed in this study suggests an alternative biological role, potentially linked to reduced invasiveness. Diener et al. [38] demonstrated that SALL4’s interaction with histone deacetylases could inhibit invasive phenotypes in melanoma, supporting the hypothesis that SALL4’s localization might influence its functional outcomes. Its lack of association with survival outcomes could be attributed to factors such as the retrospective nature of the study and the influence of other well-established prognostic factors, such as TNM stage and distant metastasis. These well-established factors often overshadow the prognostic impact of emerging biomarkers. Additionally, the retrospective nature of this study, the limited follow-up duration, and the relatively small sample size may have constrained the ability to capture the full prognostic implications of SALL4-A. With an increased number of cases and longer follow-up periods, it is possible that the prognostic significance of SALL4-A expression might become more apparent. Future studies should focus on elucidating the molecular pathways regulating SALL4-A’s cytoplasmic retention and its downstream effects in GC. Understanding these mechanisms could clarify its diagnostic specificity while revealing therapeutic opportunities.

To date, two isoforms of SALL4 (SALL4-A and SALL4-B) have been identified. Research has demonstrated that both isoforms can form complexes and engage with intracellular pathways such as TGF‐β/SMAD, Wnt/β‐catenin, HIF‐1α/VEGF signaling, PI3K/Akt, and Notch [39, 40]. However, certain studies have underscored distinct outcomes associated with the aberrant expression of SALL4 between these two isoforms. For instance, the overexpression of SALL4-B has been linked to myelodysplastic syndrome and acute myeloid leukemia in a transgenic mouse model [13]. Consequently, assessing the effects of each isoform is crucial for unraveling the roles of SALL4 in gastric tumor behavior.

Significantly, our laboratory has developed and characterized specific isoform A of anti-SALL4 monoclonal antibody (mAb) for the first time [21].To elucidate the role of the SALL4-A isoform in gastric tumor behavior, this study involved collecting formalin-fixed paraffin-embedded tissue samples from 167 gastric cancer patients, encompassing various subtypes, including signet ring cell and intestinal types. The study presents comprehensive clinical and pathological data from these samples and outlines the methodologies employed in constructing TMAs and generating a specific mAb against SALL4-A.

The assessment of SALL4-A expression at different levels in both gastric tumors and non-malignant tissue showed a significantly higher level of cytoplasmic and nuclear SALL4-A expression within gastric tumors compared to the adjacent non-malignant tissue. Accordingly, prior studies also showed SALL4 expression in GC [37, 41–43]. SALL4-A was predominantly expressed in the cytoplasm of gastric cancer cells, which correlates with favorable clinicopathological features, including lower histological grades and early-stage disease.

Although there is broad acknowledgment that the SALL4 transcription factor predominantly localizes within the cell nucleus [44], our examination revealed a distinctive pattern of SALL4-A translocation in gastric tumors. We noted an increased cytoplasmic expression of SALL4-A, surpassing the nuclear expression in these specific tumors. Cytoplasmic expression of SALL4 was also reported among other types of cancer. Lakpour et al. study showed significant expression of SALL4-A in testicular germ cell tumors [19]. Yu et al. also reported cytoplasmic expression of SALL4 in breast-invasive ductal carcinoma tissues. They observed low or undetectable expression of SALL4 in adjacent noncancerous tissues [45]. Gautam et al. also observed cytoplasmic expression of SALL4 in lung cancer tissues. SALL4 was expressed in the cytoplasm and cell membrane of lung cancer cells but not in normal or inflammatory lung tissues or normal squamous epithelium [46]. These findings suggest that aberrant expression of SALL4 in cancer tissues may lead to extranuclear expression of this marker. While the mechanisms underlying the cytoplasmic localization of SALL4-A in gastric cancer remain unclear, we hypothesize that the cytoplasmic localization of the SALL4-A isoform in GC is linked to its role in tumor progression and its interaction with various regulatory mechanisms. SALL4 has been identified as playing a crucial role in maintaining stemness, regulating angiogenesis, and modulating cancer-related pathways such as Wnt/β-catenin and VEGF signaling. Its subcellular localization can be influenced by its post-translational modifications and interaction with other proteins [36, 47].

Cytoplasmic SALL4-A may interact with cytoplasmic signaling molecules to promote oncogenic pathways, particularly by enhancing angiogenesis through VEGF regulation and by activating downstream targets associated with tumor proliferation and metastasis. These findings suggest that its cytoplasmic localization might allow SALL4-A to interact more effectively with cytoplasmic effector molecules rather than nuclear transcriptional targets.

We performed a comprehensive analysis to explore the relationship between the presence of SALL4-A in the nucleus and cytoplasm and various clinicopathologic characteristics in GC patients. To enhance clarity, we explicitly adjusted for confounders such as age, tumor size, histological grade, and TNM stage in subgroup analyses. These variables were chosen based on their established impact on GC outcomes [22, 30]. For example, higher cytoplasmic expression of SALL4-A was associated with the intestinal subtype of GC, characterized by distinct clinical behavior and prognosis. The adjustment process ensured that the observed associations were not confounded by these covariates, thus strengthening the reliability of the findings. Our findings revealed a significant correlation between increased cytoplasmic SALL4-A expression and factors such as lower histological grade and reduced TNM stage. Moreover, our subgroup analysis indicated that cytoplasmic SALL4-A expression is more commonly associated with intestinal-type GC than compared to Signet Ring Cell Carcinoma, which has a poorer prognosis. Signet Ring Cell Carcinoma is often diagnosed at more advanced stages and shows worse outcomes than non-Signet Ring Cell Carcinoma cases [48]. The association of SALL4-A expression with intestinal type aligns well with our results, suggesting that higher levels of SALL4-A are linked to less aggressive behavior in GC. Although gastric cancer includes other subtypes, insufficient sample sizes for these additional subtypes prevented their inclusion in the study. Future studies with larger cohorts may allow for a more comprehensive analysis of SALL4-A expression across all gastric cancer subtypes.

To our knowledge, apart from the present study, only one other investigation has evaluated the clinical significance of SALL4 isoform A in cancer patients, specifically within a subset of testicular germ cell tumors [19]. They found that that this isoform's higher nuclear and cytoplasmic expression was associated with worse outcomes and disease progression in testicular germ cell tumor (TGCT) subtypes, particularly seminomas and yolk sac tumors. Specifically, elevated nuclear expression correlated with advanced tumor stages in seminomas, while increased cytoplasmic expression was linked to recurrence and epididymal invasion in embryonal carcinomas​. Other studies have shown that SALL4 plays a crucial role in angiogenesis, exerting transcriptional control over VEGF expression [36]. Conversely, Diener et al. demonstrated that SALL4 negatively regulates the invasiveness of melanoma by interacting with Histone Deacetylase 2 (HDAC2) and directly binding to genes associated with invasiveness, such as Nerve Growth Factor Receptor (NGFR), E26 Transformation-Specific 1 (ETS1), Fibronectin 1 (FN1), Vascular Endothelial Growth Factor Receptor 1 (VEGFR-1), and Platelet-Derived Growth Factor C (PDGFC). Knocking down SALL4 in combination with HDAC inhibition promoted an invasive phenotype [38]. One of the significant sources of discordance in these findings may be attributed to the presence of two distinct isoforms of SALL4 (A and B), each potentially exhibiting different functions. Therefore, it is crucial to consider these isoforms, their functions, and their interactions in future investigations. Our study specifically focused on isoform "A" of SALL4, which demonstrated elevated cytoplasmic expression correlated with better prognosis of gastric tumor cells.

We examined the correlation between SALL4-A protein expression and clinical outcomes, including overall survival (OS), disease-specific survival (DSS), and progression-free survival (PFS) in gastric cancer patients. Several studies have established that elevated SALL4 expression correlates with poorer survival rates in cancer patients, though these do not specify individual protein isoforms [32, 49–51]. Notably, no existing studies have investigated patient prognosis specifically in relation to SALL4 isoforms (such as A or B) at the protein level. However, at the gene level, a study by Liu et al. explored the role of *SALL4-B* in cancer cell survival by employing targeted genetic techniques, specifically CRISPR-mediated knockdown, to selectively silence the *SALL4-B* gene in cancer cells [52]. They found that silencing *SALL4-B* alone led to a 40% increase in apoptosis, similar to the effect observed when silencing the entire *SALL4* gene. Additionally, this genetic silencing of *SALL4-B* resulted in reduced cell viability and impaired anchorage-independent growth, highlighting *SALL4-B*’s role in supporting the survival and proliferative capacity of cancer cells driven by SALL4 expression [52].

While this underscores the gene-level importance of *SALL4* isoforms in cancer progression, our study is unique in evaluating the SALL4-A isoform at the protein level, particularly in gastric cancer, which may reveal distinct biological functions that are not discernible through gene silencing alone. Further studies are warranted to clarify the specific role of *SALL4-A*, as its expression at the protein level could influence tumor behavior and patient prognosis in complementary yet distinct ways from gene-targeting approaches.

This study acknowledges some limitations. Tumor heterogeneity, a recognized challenge in Tissue Microarray (TMA) analysis, may introduce variability in antigen expression across different tumor cores. To mitigate this, we employed triplicate cores for each sample, a method shown to improve reliability and reproducibility by capturing a more representative staining pattern [24–26]. Additionally, weight loss, a common clinical feature in gastric cancer patients often linked to disease progression, was not included as a variable in this study. However, this omission is unlikely to significantly impact our findings, as the focus was primarily on the diagnostic and prognostic roles of SALL4-A expression. Similarly, family history of gastric cancer, another potential influencing factor, was not included due to the retrospective nature of the dataset and limited availability of such information. While these variables might provide additional context in future studies, their absence does not compromise the validity of the current results. Addressing these factors in subsequent research may further enhance our understanding of SALL4-A’s role in gastric cancer diagnostics and prognostics.

## Conclusion

This study highlights the expression patterns and potential clinical relevance of the SALL4-A isoform in gastric cancer (GC). Our analysis of 167 GC tissue specimens revealed a distinct cytoplasmic localization of SALL4-A in tumor cells, with significantly higher expression compared to adjacent non-malignant tissues. Elevated cytoplasmic SALL4-A expression was associated with less aggressive tumor characteristics, particularly in the intestinal subtype. However, no direct link was found between SALL4-A expression levels and patient survival outcomes. Instead, TNM stage and distant metastasis emerged as significant prognostic factors, reflecting the multifactorial nature of GC prognosis.

These findings suggest that cytoplasmic SALL4-A expression may have diagnostic value in differentiating malignant from non-malignant tissues in GC. Further research, including larger, more diverse cohorts and mechanistic studies, is needed to better understand the functional role of SALL4-A and to evaluate its potential clinical applications. This work contributes to the growing body of knowledge on GC biomarkers but underscores the need for cautious interpretation and additional validation before considering clinical translation.

## Data Availability

No datasets were generated or analysed during the current study.
